# Partial-Methylated HeyL Promoter Predicts the Severe Illness in Egyptian COVID-19 Patients

**DOI:** 10.1155/2022/6780710

**Published:** 2022-05-31

**Authors:** Hewida H. Fadel, Mohammad Abd EL-Rahman Ahmed, Kareem Mahamoud Gharbeya, Mohammed Ahmed Khamis Mohamed, Mohamed N. Roushdy, Reda Almiry

**Affiliations:** ^1^Department of Medical Laboratory Technology, Faculty of Allied Medical Science, Pharos University, Alexandria, Egypt; ^2^Department of Clinical Pathology, Military Medical Academy, Alexandria Armed Forces Hospital, Egypt; ^3^Critical Care Consultant at Alexandria Armed Forces Hospital, Egypt; ^4^Pulmonary Consultant at Alexandria Armed Forces Hospital, Egypt; ^5^Department of Medical Microbiology and Clinical Immunology, Military Medical Academy, Alexandria Armed Forces Hospital, Egypt; ^6^Molecular and Diagnostic Microbiology, Molecular Microbiologist Consultant at Alex Armed Forces Hospital, Egypt

## Abstract

**Background:**

To date (14 January 2022), the incidence and related mortality rate of COVID-19 in America, Europe, and Asia despite administrated of billions doses of many approved vaccines are still higher than in Egypt. Epigenetic alterations mediate the effects of environmental factors on the regulation of genetic material causing many diseases.

**Objective:**

We aimed to explore the methylation status of HeyL promoter, a downstream transcription factor in Notch signal, an important regulator of cell proliferation and differentiation blood, pulmonary epithelial, and nerves cells.

**Methods:**

Our objective was achieved by DNA sequencing of the product from methyl-specific PCR of HeyL promoter after bisulfite modification of DNA extracted from the blood samples of 30 COVID-19 patients and 20 control health subjects and studying its association with clinical-pathological biomarkers.

**Results:**

We found that the HeyL promoter was partial-methylated in Egyptian COVID-19 patients and control healthy subjects compared to full methylated one that was published in GenBank. We identified unmethylated CpG (TG) flanking the response elements within HeyL promoter in Egyptian COVID-19 patients and control healthy subjects vs. methylated CpG (CG) in reference sequence (GenBank). Also, we observed that the frequency of partial-methylated HeyL promoter was higher in COVID-19 patients and associated with aging, fever, severe pneumonia, ageusia/anosmia, and dry cough compared to control healthy subjects.

**Conclusion:**

We concluded that hypomethylated HeyL promoter in Egyptian population may facilitate the binding of transcription factors to their binding sites, thus enhancing its regulatory action on the blood, pulmonary epithelium, and nerves cells in contrast to full methylated one that was published in GenBank; thus, addition of demethylating agents to the treatment protocol of COVID-19 may improve the clinical outcomes. Administration of therapy must be based on determination of methylation status of HeyL, a novel prognostic marker for severe illness in COVID-19 patients.

## 1. Introduction

To date, the prevalence, incidence, and mortality rate in America, Europe, Russia, Australia, and Asia were very higher than in Egypt [1]. The aggressiveness and severity of SARS-CoV-2 and its variants is still life-threatening despite 2 years of global efforts using all the world facilities: quarantine, numerous treatment protocols, and administration of billion doses of vaccines, especially in developed countries [2, 3]. All of these paid our attention towards the possible role of genetic, epigenetic, and environmental factors that may mediate the immune response against SARS-CoV-2, causing the noticed difference between the prevalence and mortality rate of COVID-19 in Egypt from other countries. Nevertheless, rare studies explain the molecular mechanism of its infectivity and virulence. Of note, the severe illness and various symptoms that involved all human organs causing multiple organs disorders (MOD) and failure, especially thrombosis and hemorrhagic stroke, supported the involvement of the blood disorders in the severity of COVID-19 due to the dysregulation of hematopoiesis accompanied with cytokine storm, which influences the whole body causing pulmonary, vascular, and nervous disorders [4]. As known, lung plays an important role in platelets biogenesis. Among many cellular and molecular components that participate in immune response against SARS-CoV-2, platelets and their surface proteins include membrane associated-glycoproteins and -cytokines [5]. Hey, an effector transcription factor in Notch signal, is encoded by a family of genes Hairy and enhancer-of-split-related with a YRPW motif (Hey) family of genes (Hey-1, Hey-2, and Hey-L). HeyL is located on chromosome 1 (GRCh38.p13), spans 16209 bp, and consists of 5 exons and 4 introns. HeyL plays important roles in differentiation of blood, cardiac, neural, and vascular progenitor cells. Of importance, it mediates megakaryopoiesis, preferred the platelets production, and shifts the differentiation of participate in the regulation of vascular and pulmonary homeostasis [6, 7]. Many infectious and noninfectious diseases such as asthma might alter the epigenetic processes which in turn influence the immune response, eliciting the inflammation process. Epigenetics changes mediate the lung dysfunction where hypo or hypermethylation usually results in disturbance of gene expression and is strongly associated with age-related diseases including cancer, atherosclerosis, and cardiovascular diseases [8]. Of importance, it has been demonstrated that term hyper or hypomethylation is relative, especially, a lot of evidence indicate the polymorphic variations in the methylation status between populations [9]. In our study, we used terms partial-methylated for those HeyL promoter showing mixed bands (methylated and unmethylated). Thus, we aimed to evaluate the role of the methylation status of HeyL gene and its relation to the clinical findings of COVID-19 including pneumonia, pulmonary embolism, dry cough, diarrhea, and anosmia and ageusia via monitoring the routine proinflammatory biomarkers including C-reactive protein (CRP), ferritin, and D-dimer.

## 2. Materials and Methods

The present study was performed in Armed Forces Hospital in Alexandria, Egypt, during the first wave of COVID-19 outbreak. The blood samples were collected from 30 inpatients who were diagnosed with COVID-19 by a real-time polymerase chain reaction (RT-PCR) upon admission in the hospital and 20 health control. All subjects signed a written informed consent before participation and the study was approved by the Ethics Committee of the Medical Research Institute (IORG 0008812). Chest CT scan was done for all inpatients while Angiography CT scan was done for those expected to have thromboembolism. Patients who had SPO2 less than 85% were admitted to the intensive care unit (ICU). The data in our study are based on the clinical and biochemical investigations for COVID-19 patients upon admission in the hospital and follow-up of the clinical registry to monitor the clinical outcomes. Extraction of DNA from whole blood samples is used for investigation of epigenetic modifications of HeyL gene. Data collected from registration include demographic characteristics features, complete blood count, and inflammatory markers C-reactive protein (Crp), ferritin, and D-dimer.

### 2.1. Statistical Analysis of Published Data on WHO Website

We analyzed the data published by the WHO including the prevalence, incidence, and deaths related to COVID-19 by the end of 2020 (time of our study) and the recent situation (mid of January 2021) in Egypt compared to the global situation.

### 2.2. Genomic DNA Extraction and Bisulfite Modification

Genomic DNA was extracted using a QIAamp DNA Mini kit (Qiagen, Valencia, CA, USA). The purity of products was quantified by a nanodrop spectrophotometer at 260/280 nm (Jenway) before treated with sodium bisulfite using an EpiJET Bisulfite Conversion kit (Thermo Fisher Scientific) (Catalogue #K1461). The bisulfite modification was based on conversion of the unmethylated cytosines to uracils while the methylated cytosines were protected from sulfonation. Add 120 *μ*L of modification reagents to 20 *μ*L of DNA sample containing 400-500 ng of purified genomic DNA into a PCR tube, mix, and centrifuge the sample to the bottom. Place PCR tubes into the thermal cycler (98°C for 10 min, 60°C for 150 min). Add 400 *μ*L of binding buffer, centrifuge 12,000 rpm, and discard the flow-through. Add 200 *μ*L of wash buffer, centrifuge 12,000 rpm, and discarded the flow-through. Add 200 *μ*L of desulfonation buffer and let to stand at room temperature for 20 min. Wash twice with 200 *μ*L wash buffer. Finally, add 10 *μ*L of elution buffer to the microcolumn and centrifuge 12,000 rpm for 60 sec. We eluted purified DNA in a total volume of 50 *μ*L TE Buffer.

### 2.3. Methyl-Specific PCR (MSP)

The methylation status of CpG in promoter of HeyL gene was evaluated by methyl-specific PCR (MSP) using a primer set for the MSP; Methylated Forward:5′-TTTTATAGGTTAGTAGCGTTTAGGC-3′, Methylated reverse:5′-GACTCTACTCCGCTATCTCGAC-3′,Unmethylated forward F:5′-TTTATAGGTTAGTAGTGTTTAGGTGA-3′,Unmethylated reverse: 5′-TCCAACTCTACTCCACTATCTCAAC-3′. The HeyL promoter has been described in a previous study by using the upstream promoter area for the amplification of CpG islands containing promoter DNA sequences of either unmethylated (UMSP) or methylated (MSP) DNA sequences. PCR amplification was performed as described previously [10]. 25 *μ*l of the PCR reaction contained 1 *μ*l methylated primers (forward), 1 *μ*l methylated primer (reverse), 1 *μ*l of bisulfite-treated DNA, U JumpStart Taq polymerase (Sigma-Aldrich, St. Louis, MO, USA), and 22 *μ*l deionized water (the same for unmethylated primers). The PCR conditions were follows: 95°C for 3 min, 40 cycles (95°C for 30 sec, 54°C for 30 sec, and 72°C for 1 min), and 72°C for 5 min. A 10 *μ*L sample of each PCR product was mixed with 1× loading buffer and analyzed by electrophoresis on nondenaturing 2% agarose gel stained with ethidium bromide and visualized on UV. Direct sequencing of PCR products was performed by LGC genomic sequencing service at Biosearch Technologies (Germany). Sequencing runs have been performed on 3730xl DNA Analyzer [Applied Biosystems™] using POP-7™ Polymer BigDye™ [Applied Biosystems™]. Sequencing chemistry was DyeTerminator v3.1 Cycle Sequencing Kit [Applied Biosystems™]. The data was provided in the form of chromatogram.

### 2.4. Bioinformatic Analysis of HeyL Promoter

To compare the sequences obtained from chromatogram with those in reference sequence, we convert it into FASTA format using EMBOSS Seqret and Clustal Omega to align with reference sequence and determine the binding sites of TFs among the target sequence. Then, we used Quma [11], a quantification tool for methylation analysis, to compare the methylation status between COVID-19 patients and control subjects with the genome sequence obtained from GenBank. By using Ensembl program [12], we detect the transcription start site (TSS) 5′-AGGCAGCCTG-3′ and seven binding sites for transcription factors (TFs) including 5′-AGGCC-3′, a consensus sequence of Poly (ADP-ribose) polymerase (PARP-1), 5′-[GA][CA]GACCC-3′, a consensus sequence of zinc finger protein response element (FRE) zinc finger and BTB domain-containing protein 7A (ZBTB7A), 5′-CANNTG-3′, a consensus sequence of USF, an upstream stimulated factor which binds to E-Box (*N* = any nucleotide),5′-RRKNSA-3′, a consensus sequence of vitamin D receptor (VDR), where *N* is equal to any nucleotide, *R* is equal to *A* or *G*, *K* is equal to *G* or *T*, *S* is equal to *C* or *G*, CAGA box 5′- AGAC-3′, in COVID-19 patients and compared its methylation status with reference sequence in GenBank and control subjects.

### 2.5. Statistical Analysis

Statistical analysis was performed using SPSS 15.0 statistical software. Odd ratio (OR) was used to calculate the ratio of the odds and 95% confidence interval (CI) of an event occurring in a risky group to the odds of it occurring in the nonrisky group. *Z* test was used for analytic comparison. All quantitative data are presented as the mean ± SE. We used Student's *t*-test for quantitative variables to compare between the two studied groups. *P* ≤ 0.05 was considered significant.

## 3. Results

### 3.1. Statistical Analysis of Published Data on WHO Website

**Figure 1:**
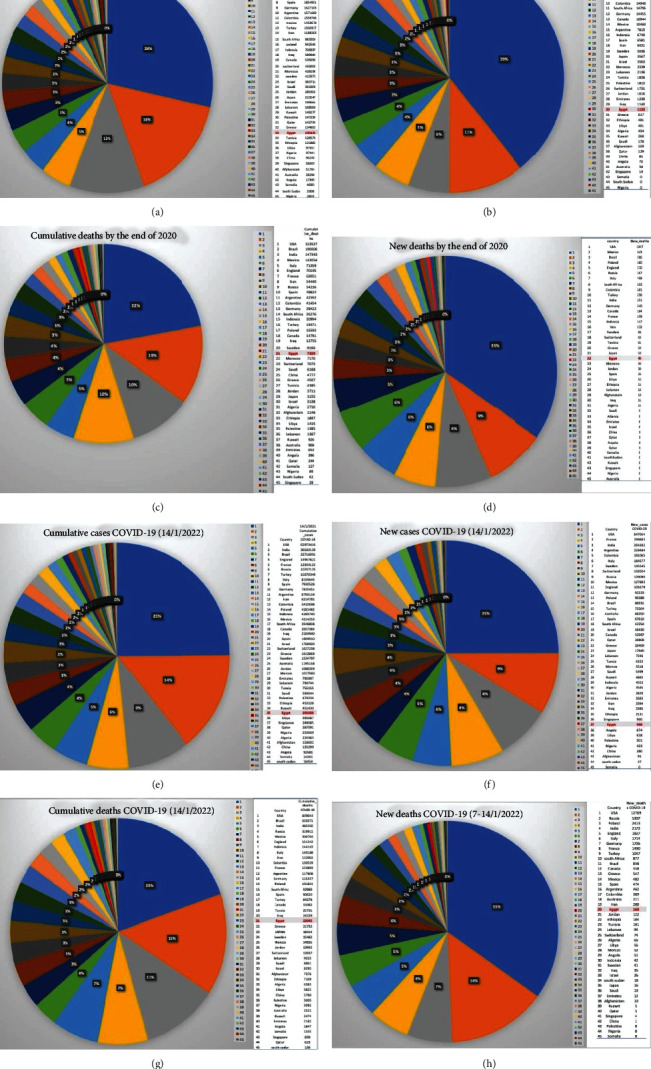
Data analysis of the COVID-19 prevalence (a and e), incidence (b and f), and deaths (c, d, g, and h) in Egypt and generally in Africa by the end of first wave (Dec. 2020) to date (mid-Jan. 2022) compared to that in Americas, Europe, Asia, and Australia. (The data was for 45 chosen countries that display the most population worldwide and published by the WHO [1]), Egypt is shaded by red color.

Analyzing the published data of COVID-19 pandemic on WHO website showed that the prevalence, incidence, and its related mortality rate of COVID-19 in Egypt and generally in Africa are very lower than in America, Europe, Asia, and Australia by the end of 2020 before the approval of many vaccines. Surprisingly, to date and after administration of billions doses of approved vaccines, especially, in developed countries, the prevalence of SARS-CoV-2 variants and related death is still significantly higher than in Egypt (Figure 1, Supplementary file 1). We ranked 45 countries which represent the most population of the worldwide, we found that the prevalence, incidence, and mortality rate were very low compared to the countries in America, Europe, Asia, and Australia in 2020 as well as in the current month Jan. 2022. The order of Egypt prevalence, incidence, mortality rate, and new death were 33^rd^, 30^th^, 21^st^, and 22^nd^ by the end of 2020 and the same pattern 35^th^, 37^th^, 21^st^, and 20^th^ was observed in the current month Jan. 2022. As data has shown, to date, the spreading and death related to COVID-19 pandemic have the same pattern.

### 3.2. The Frequency of Methylated HeyL Promoter

**Figure 2:**
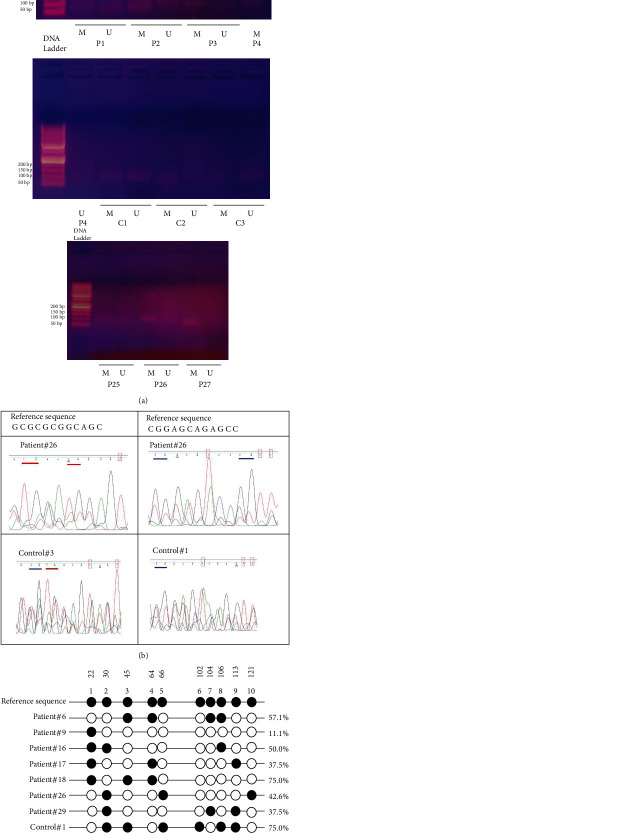
(a) PCR products were separated using 2% agarose gel electrophoresis of methyl-specific PCR amplified region of HeyL promoter in Egyptian COVID-19 patients (#1, #2, #3, #4 #25, #26, and #27) vs. control subjects (#1, #2, and #3), (b) Sequencing chromatogram images of DNA sequence of HeyL promoter showing mCpG (blue underlined), unmethylated CpG (red underlined), and unmethylated cytosine (red box) in HeyL promoter in P#26 and C#1, and C#3 compared to the references sequence. (c) Lollipop presentation of methylation rate of HeyL promoter in Egyptian COVID-19 patients and control subjects compared to the reference sequence (GenBank), mCpG (black circle), and TG (white circle).

Electrophoretic separation of amplicon products from methyl-specific PCR showed that visible bands for PCR products corresponding to amplicon products which were amplified by methylated primers in COVID-19 patients cases were stronger than that were amplified by unmethylated primers (P#2, #3, #4, #25, #26, and #27) while visible bands corresponding to products from unmethylated primers in control healthy subjects as in C #1 and #3 were stronger than that were amplified by unmethylated primers (Figure 2(a). Statistical analysis of our data showed that partial-methylated (strong methylated and faint unmethylated bands) HeyL promoter was significantly more frequent in COVID-19 patients 22 out of 30 (74.2%) compared to control healthy subjects 9 out 20 (26.3%), (OR = 3.3611 with confidence interval (CI 95% 1.0162-11.117) *P* = 0.047 as shown in Table 1.

### 3.3. Bioinformatic Analysis of HeyL Promoter

Bioinformatic analysis of data obtained from Sanger sequencing of HeyL promoter in COVID-19 patients and control subjects helped us to evaluate the methylation status of target sequence including methylated cytosine either CpG (mCpG) or non-CpG (mC) as well as the location of methylated cytosine within HeyL promoter in comparison to the reference sequence published in GenBank under the accession number (Primary (citable) accession number: Q9NQ87, Secondary accession number(s): Q5TG99, Homo sapiens chromosome 1, GRCH38.P13, cover region: 39623435-39639643, Entrez gene ID: 26508) [13]. We observed that the methylation status of HeyL promoter in Egyptian COVID-19 and control healthy subjects was lower than that in the published reference sequence. Also, we found that the mCpG rates were ranged from 11.15% to 75.0% in COVID-19 patients vs. 14%-75% in control healthy subjects compared to full methylated HeyL promoter in reference sequence (Figures 2(b) and 2(c)).

**Figure 3:**
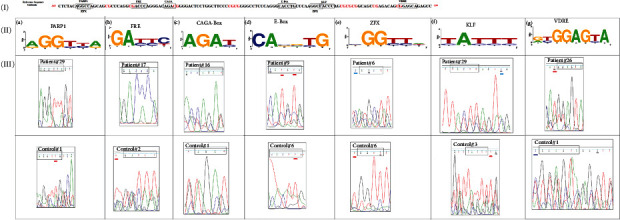
**(**I**)** Reference sequence (Genbank) span from -239 to -365 upstream TSS; (II**)** logos sequencing analysis of transcription factors binding sites in the HeyL promoter for samples obtained from COVID-19 patients and control subjects compared to the consensus sequence in reference sequence showing the amount of information at each position in each motif at *y*-axis for (a) PARP1 binding motif, (b) FRE binding motif, (c) CAGA-Box binding motif, (d) E-Box binding motif, (e) ZFX binding motif, (f) KLF binding motif, and (g) VDRE binding motif; (III**)** comparison between the methylation status of binding sites in COVID-19 patients (upper) and control healthy subjects (lower). Symbol of Nucleotides: G = guanine; A = adenine; C = cytosine; T = thymine; R = purine (A or G); Y = pyrimidine (C or T); N = any nucleotide Methylated cytosine CpG (blue underlined), unmethylated cytosine (TG) (red underlined).

To investigate the effect of methylation status on the HeyL promoter action, we compared the binding sites in both COVID-19 patients and control with those in reference sequence using Ensembl program. We found that mCpG flanking 3 out 7 of binding sites in the reference sequence; FRE, CAGA box, and VDRE. In Egyptian COVID-19 patients, methylated cytosines either CpG or non-CpG were more frequent flanking these binding sites than in control subjects. In addition, we identified unmethylated and partial-methylated KLF binding sites among HeyL promoter in COVID-19 patients vs. the consensus sequence of KLF (CACCC). Surprisingly, we identified methylated E-Box in COVID-19 patients CACGTG vs. CACCTG in reference sequence. Collectively, HeyL promoter in Egyptian population either COVID-19 patients or control healthy subjects was hypomethylated compared to the reference sequence published in GenBank (full methylated); however, partial methylated HeyL promoters containing methylated cytosine either CPG or non-CpG in Egyptian COVID-19 patients were more frequent than in control healthy subjects who showed more unmethylated ones as shown in Figure 3.

### 3.4. Effect of mCpG Flanking Binding Sites of HeyL on the Clinical-Pathological Features of COVID-19

**Figure 4:**
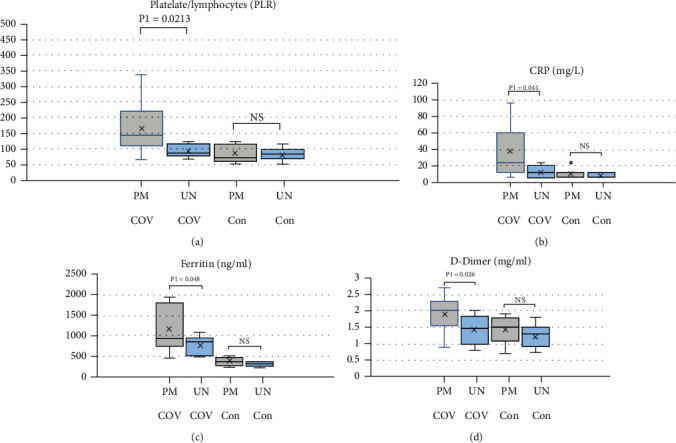
Box plot presents the effect of methylated status of HeyL promoter on the proinflammatory biomarkers including (a) platelet-to-lymphocyte ratio (PLR), (b) CRP, (c) ferritin, and (d) D-Dimer in COVID-19 patients compared to control subjects. Partial-methylated HeyL promoter (grey box) and unmethylated HeyL promoter (blue box). The significance level was considered as *P* < 0.05. PM: partial-methylated; UM: unmethylated; COV: COVID-19; Con: control subjects; NS: insignificant difference.

As shown in Table 2 the clinical-pathological features of COVID-19 patients were categorized according to methylation status of HeyL promoter into partial-methylated and unmethylated. In the present study, COVID-19 patients had a mean age 47.6 ± 11.22 and comprised of 18 out of 30 (60%) males and 12 females (40%). Of note, partial-methylated HeyL promoter was more frequent in aging COVID-19 patients who were equal or more than 50 years old compared to those who were less than 50 years old (*P* = 0.0158). We found that partial-methylated of HeyL promoter increased the risk of COVID-19 in aging patients older than 50 years old 10.2 times than patients who were less than 50 years old. However, the frequency of partial-methylated promoter of HeyL gene did not show significant difference among COVID-19 patients in relation to sex. Also, we found that partial-methylated of HeyL promoter was associated with severe illness in COVID-19 patients. Statistical analysis of our data showed that partial-methylated of HeyL promoter increased the risk of severe pneumonia 5.66 times (*P* = 0.0513), fever 10 times (*P* = 0.0246), anosmia/ageusia 7.5 times (*P* = 0.0278), and dry cough 10.55 times (*P* = 0.0140). Assessment of proinflammatory biomarkers showed that partial-methylated HeyL promoter was associated with the significant elevation of routine proinflammatory markers including platelet to lymphocytes (PLR) (*P* = 0.0213), CRP (*P* = 0.044), ferritin (*P* = 0.048), and D-dimer (*P* = 0.026) compared to those who showed unmethylated HeyL promoter in COVID-19 patients Table 3, Figure 4).

**Figure 5:**
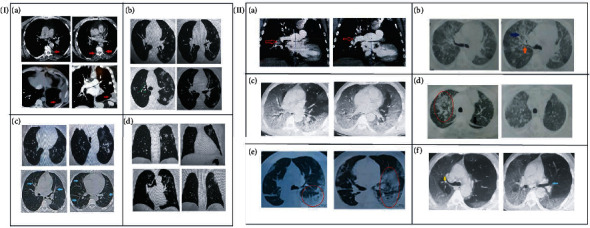
(I) Computerized tomography (CT) axial image of COVID-19 patients had moderate illness showing (a) plural effusion at the lower lobe (red arrow); (b) faint ground-glass opacity (GGO) and multiple nodular (green arrow); (c) tree-in-bud pattern (turquoise arrow), bronchiectasis, and air trapping; and (d) coronal image showing plural effusion. (II) Computerized tomography (CT) angiography coronal image of COVID-19 patients who suffered from severe illness showing (a) pulmonary embolism in the right pulmonary artery (red arrow); (b) axial image showing fibrotic bronchiectasis (blue arrow) and nodular air bronchogram (orange arrow); (c) diffuse massive bilateral GGO with tree-in-bud pattern; (d) massive consolidation (red circle) with bands in the lower right and left lobes (black arrow); (e) massive consolidation (red circle) in the left lobe; and (f) diffuse GGO in the right lobe and pulmonary nodules (yellow arrow) and air bronchogram (blue arrow).

Angiography CT for the chest of COVID-19 patients showed that the most common clinical findings were varied from peripheral ground-glass opacities (GGO), plural effusion, and centrilobular nodules with a linear branching forming tree-in-bud pattern in moderate cases (Figure 5(i)) which was extended to diffused, patchy, bilateral form, and linear opacities forming band-like GGO in COVID-19 patients who suffered from severe illness. In addition, air bronchogram, consolidation, traction bronchiectasis, crazy paving pattern, and plural effusion were observed in critical cases who admitted intensive care unit (ICU) as shown in (Figure 5, II).

## 4. Discussion

The published data by the WHO from the beginning of COVID-19 pandemic among 2020 indicated that its prevalence, incidence, and related mortality rate in America, Europe, Asia, and Australia are significantly higher than in Egypt and Africa. To date, all variants (Beta, Delta, and Omicron) are spread in the same pattern [14]. Progressive spreading of SARS-CoV-2 variants despite administration of billion doses of vaccines indicates the importance of drug repositioning of approved therapies. This paid our attention towards the possible role of epigenetic alterations in the observed gap. Thus, we need more researches to explore more target for individual therapies based on determination of specific biomarker which plays an essential role in regulation of immune response via regulation of cell proliferation and differentiation. Of these, HeyL plays an important role in the cell proliferation and differentiation of blood, cardiac, neural, pulmonary epithelial, and vascular progenitor cells. Thus, we aimed to monitor its methylation status and its impact on the response elements within HeyL promoter in COVID-19 patients and, in addition to, study its correlation with the clinical outcomes and the routine biomarkers including PLR, CRP, ferritin and D-dimer.

As mentioned in the most published articles, all blood cell lineages mediate the SARS-CoV-2-related severity and death. Although SARS-CoV-2, RBCs, and platelets lack nucleus, they have their own translation machine including mRNAs, miRNAs, TFs, and proteins may interact with human translation machine in such way that manipulate the cell cycle and homeostasis. For example, it was found that Coronavirus silenced the cell cycle at S phase [15, 16]. In this aspect, mRNAs and miRNAs enable in RBCs and platelets to synthesize protein and regulate their function including response to oxidative stress and iron availability as in case of SARS-CoV-2-induced hypoxia and inflammation [17]. While blood cell biogenesis is carried out mainly in the bone marrow, WBCs and platelets have other pathways in the thymus and lung, respectively, the same targets for SARS-CoV-2 infection. Thus, methylation status of human genome may mediate the infectivity action of SARS-CoV-2 as well as the response of host cells. However, a rare study evaluated the role of hypermethylation of angiotensinogen activating enzyme (ACE2) in aging people increase the susceptibility to SARS-CoV-2 infectivity and severity by facilitating T cell viral infections and distribution [18].

In electrophoretic separation, the amplicon products of MSP showed that partial-methylated HeyL promoter (methylated+unmethylated bands) among COVID-19 patients was more frequent compared to control subjects who showed more unmethylated bands. It is well known that hypermethylation of promoters within human genome is considered an epigenetic clock that may be a predictor of the biological age and many diseases such as autoimmune disease and cancers [19]. Two proposed mechanisms of this relationship are as follows: first, hypermethylation of promoter exerts inhibitory effect on the gene expression via interfering with binding of TFs; second, recruiting repressors to their consensus sequences within the regulatory region [20]. First of all, a previous study pointed to the role of partial-methylation in downregulation of HeyL gene [10]. As known, aberrant methylation of TFs such as HeyL is responsible for the regulation of the differentiation of blood and pulmonary cells causing raising the ratios of platelet/lymphocyte ratio as well as goblet/secretory cells, respectively, resulting in disturbance of blood and pulmonary cell homeostasis and decreasing the mucus secretion causing dry cough.

Also, we found that binding sites in Egyptian HeyL promoter were hypomethylated than those in reference sequence; however, the methylation status of binding sites in COVID-19 patients was higher than control healthy subjects. These binding sites depend mainly on zinc to bind with DNA such as PARP-1 [21] and ZBTB7A [22] which has two homologous zinc finger domains while KLF [23] has three C2H2 zinc finger domains. Also, E-Box can bind to and regulated by zinc finger proteins as well as USF [24]. Moreover, two zinc finger-like structures present and mediate the binding of vit-D receptor to VDRE in target genes [25].

All of these support the importance of zinc and vit-D in the treatment of COVID-19 [26]. Particularly, vit-D deficiency due to aging impairs the muscle regeneration via reducing the proliferative ability of Notch/Hey pathway [27]. Thus, we may suggest that addition of demethylating agents such as Azacytidine plus zinc and vit-D will help in facilitating the binding of TFs to their binding sites, subsequently improving the regulatory actions of HeyL on the blood, pulmonary epithelium, olfactory nerves, and muscle cells. Especially, these TFs play different important roles in the regulation of immune response. Particularly, a recent study indicated the efficacy of demethylating agents in treatment of viral infection [28].

For instance, PARP-1 is the dominant transcription regulator of viral infection, replication, and virulence where it has been implicated in the virus-induced inflammation via regulating the expression of cytokine production [29]. A zinc finger protein (ZNF) is the largest class among about 2000 site-specific DNA-binding TFs in the human genome. Of these, ZFX is a transcriptional activator that has been implicated in the enhancing of cell cycle progression [30]. ZBTB7A suppresses the gene expression of many target genes. For example, it inhibits the TGF-beta/Smad signal transduction. Also, it shifts the lymphoid progenitors' differentiation towards B cells rather T lineages via inhibiting the expression of Notch and their downstream genes including Hey family [31]. Also, CAGA motif is the main binding element for Small mothers against decapentaplegic (SMAD)2/3/4 which are families of TFs that regulate diverse processes in innate and adaptive immune responses including driving of antiviral responses and proinflammatory responses and regulating immune cell differentiation. It was revealed that Smad mediated the inhibitory action of HeyL on transforming growth factor-*β-* (TGF-*β-*) induced cell cycle arrest. Our data reveal that the presence of response elements to Smad binding site within HeyL promoter may mediate their interaction with SARS-CoV-2, affecting the viral spreading and cell migration. Moreover, our data may suggest that the presence of methylated E-Box in HeyL promoter may interfere with the binding of USF-1, subsequently impairing its regulatory action on the lung-specific glycoprotein, inflammatory process, and the pulmonary epithelium, especially, during viral infection [32]. In addition, KLF2 and KLF4 play an essential role in maintaining endothelial barrier integrity, homeostasis, regulation of macrophage M1/M2 polarization, B-cell differentiation, maintaining vascular health, and enhancing the anti-inflammatory process via regulating TGF-*β* and platelet-derived growth factor (PDGF) [33]. Subsequently, our finding may emerge a novel role of HeyL in atherogenesis related to COVID-19 patients via mediating the action of KLF signal.

The association between the high frequency of partial-methylated HeyL promoter on the clinical-pathological findings in COVID-19 patients was clearly observed as the worst clinical outcomes including severe pneumonia, fever, dry cough, and ageusia and anosmia. In this aspect, the roles of Notch/Hey signal pathway in the regulation of cell proliferation and differentiation in lung, myogenesis, hematogenesis, cardiovascular system embryo neurogenesis, and mature olfactory sensory neurons were well established [34]. We may suggest that partial-methylated of HeyL promoter resulted in shifting the differentiation of pulmonary epithelial cells towards ciliated cells causing the dry cough as well as shift erythroid/megakaryocyte differentiation towards platelets eliciting the ratio of platelets to lymphocytes (PLR) and proinflammatory markers including CRP, ferritin, and D-dimer, resulting in the observed fever, dry cough, and severe pneumonia in COVID-19 patients. In consistent with our data, many studies indicated the hypermethylation in aging people; subsequently, our finding may interpret the worst clinical outcomes and multiple organ disorders (MOD), especially, in aging COVID-19 patients. Consistently, a previous study reported that 20% of COVID-19 patients with poor prognosis had had inflammatory and thrombotic process-related symptoms such as coagulopathy and pleural effusion as a result of cytokines storm induced by SARS-COV-2 [35]. Recent study supports our finding in that SARS-CoV-2 proteins interact with and affect on the molecular machinery of human pulmonary ciliated epithelial cells causing loss of their integrity via production of abnormal cytoskeleton proteins within few days (3-7) postinfection [36]. Also, a recent study remarks that the COVID-19 symptoms may last 3 to 12 months, approximating the lifespan of ciliated epithelial cells in the lung which is ranged from 6 to 8 months [37]. The observed poor prognosis in COVID-19 patients who showed severe illness may be attributed to the improper binding of regulatory TFs to their response elements in HeyL promoter, subsequently, interfering with the regulatory role of HeyL. Our data revealed that methylated HeyL promoter may be a prognostic marker for severe illness in COVID-19 patients.

Previously, we may suggest that the high frequency of partial-methylated HeyL promoter in aging patients has been implicated in the susceptibility to SARS-CoV-2 and its related severe illness including dry cough, anosmia and ageusia, and severe pneumonia. Also, our data indicated that the relevance of detecting the methylation status of HeyL promoter did not have only a prognostic value, but also being as a target for novel antiviral therapies which may add benefits in counteracting the severe illness in COVID-19 patients. For example, demethylating agents such as 5-azacytidine or natural methylation regulators such as lycopene may help in the treatment of COVID-19 patients; especially, their efficacy in the treatment of asthma was investigated [38].

Finally, we postulate that hypomethylation of HeyL promoter may exert a protective effect in Egyptian people by enhancing the gene expression of HeyL, a target TF in Notch signal which participates in the regulation of numerous pathways including cell proliferation and differentiation. Also, we proposed a mechanism for the global aggressiveness of COVID-19 pandemic especially among elderly that the long-term exposure to any environmental risk factors especially in aging people caused epigenetic alterations such as hypermethylation of DNA making them vulnerable to SARS-CoV-2 infectivity and severity. As known, aging was itself considered a disease and strongly associated with many comorbidities that contributed to severe illness related with COVID-19 [39]. Concurrently, we found that the mean age of and mortality rate in Egyptian COVID-19 patients were lower than that in many published data from Western countries [40, 41]. Of course, high income, social insurance, and well-developed state medical care support programs have an implication in the high life expectancy in Western countries comparing to that in Arab countries [42].

To our knowledge, our work may present a novel approach in interpretation of the severity of COVID-19 in aging patients and it is the first time to compare the methylation status of HeyL promoter in Egyptian population in either COVID-19 patients or healthy subjects with that published in GenBank HeyL.

## 5. Conclusion

We concluded that hypomethylated HeyL promoter in Egyptian population is considered a protective factor while hypermethylation may impair the regulatory action of HeyL via interfering with binding of TFs to their binding sites. Thus, determination of the methylation status HeyL promoter can predict not only the clinical outcomes but also a target of demethylating agents.

## Figures and Tables

**Table 1:** The frequency of HeyL promoter methylation status in COVID-19 patients compared to that in the control group.

	COVID-19 patients *n* = 30 *N* (%)	Control *n* = 20 *N* (%)	Odd ratio (OR)	95% CI	*Z*	*P* value
Partial-methylated	22 (73.3)	9 (45)	3.3611	1.0162-11.117	1.986	0.047
Unmethylated	8 (26.6)	11 (55)				

A standard normal deviate (*z*-value) is calculated as ln(OR)/ynn{ln(OR)}. CI: confidence interval. *P* value for comparing between the frequency of partial-methylated and unmethylated promoter of HeyL gene among COVID-19 patients and the control group. Statistically significant at *P* ≤ 0.05.

**Table 2:** Correlation between methylation status of the promoter of HeyL gene and clinical-pathological features in COVID-19 patients.

Clinical-pathological characteristic features	Partial-methylated (*n* = 22)	Unmethylated (*n* = 8)	Odd Ratio (OR)	95% CI	*Z*	*P* value
Sex						
Male (%)	14 (63.6)	5 (62.5)	1.05	0.1968-5.6021	0.057	0.9545
Female(%)	8 (36.3)	3 (37.5)				
Age						
(≥50)(%)	17 (77.2)	2 (25)	10.20	1.5478-67.2189	2.414	0.0158^∗^
(<50)(%)	5 (22.7)	6 (75)				
Fever (>38.5°C) (%)	20 (90.9)	4 (50)	10.00	1.3420-74.5140	2.247	0.0246^∗^
Fever (37°C-38.5°C)(%)	2 (9.09)	4 (50)				
Severe pneumonia (%)	17 (77.2)	3 (37.5)	5.66	0.9902-32.4293	1.949	0.0513^∗^
Mild/moderate pneumonia (%)	5 (22.7)	5 (62.5)				
Anosmia/ageusia (%)	18 (81.8)	3 (37.5)	7.50	1.2457-45.1542	2.200	0.0278^∗^
No anosmia/ageusia (%)	4 (18.2)	5 (62.5)				
Pulmonary embolism (%)	7 (31.8)	0 (0)	8.23	0.4168-162.351	1.38	0.1661
No embolism (%)	15 (68.2)	8 (100)				
Dry cough (%)	19 (86.3)	3 (37.5)	10.55	1.6119-69.1239	2.458	0.0140^∗^
Mucous (%)	3 (13.6)	5 (62.5)				
No diarrhea (%)	10 (45.4)	4 (50)	0.83	0.1649-4.2118	0.221	0.8254
Diarrhea (%)	12 (54.5)	4 (50)				
ICU admission (%)	8 (36.3)	2 (25)	1.714	0.2775-10.5898	0.580	0.5618
No ICU admission (%)	14 (63.6)	6 (75)				

A standard normal deviate (*z*-value) is calculated as ln(OR)/SE{ln(OR)}. CI: confidence interval. *P* value for comparing between the clinical pathological findings and routine biomarkers of COVID-19 patients regarding to the methylation status; partial-methylated and unmethylated HeyL promoter. Statistically significant at *P* ≤ 0.05.

**Table 3:** Comparison between the mean level of inflammatory markers and methylation status of the promoter of HeyL gene in COVID-19 patients.

	COVID-19 patients			Control subjects		
Proinflammatory markers	Partial-methylated (*n* = 22)	Unmethylated (*n* = 8)	*P*1 value	Partial-methylated (*n* = 16)	Unmethylated (*n* = 4)	*P2* value
PLR	177 ± 95.31	93.3 ± 19.05	0.021	84.77 ± 27.22	81.83 ± 12.61	0.76
CRP (mg/L)	38.18 ± 33.62	12.75 ± 6.99	0.044	10.6 ± 5.49	7.63 ± 2.67	0.133
Ferritin (ng/ml)	1171.8 ± 541.22	761.25 ± 223.27	0.048	368.7 ± 92.18	309 ± 47.02	0.077
D-dimer (*μ*g/ml)	1.9 ± 0.51	1.43 ± 0.41	0.026	1.42 ± 0.38	1.22 ± 0.31	0.21

*P*1: significant value for comparing between the mean levels of inflammatory markers in COVID-19 patients according to methylation status of HeyL promoter. *P*2: significant value for comparing between the mean levels of inflammatory markers in control subjects according to methylation status of HeyL promoter. Statistically significant at *P* ≤ 0.05.

## Data Availability

The data used to support the findings of our study are available from the corresponding author upon request.

## Supplementary Materials

Supplementary Materials Supplementary 1: Powerpoint showed the presentation of the analysis of data published by the WHO [1] of the prevalence, incidence, and deaths related to the spreading of COVID-19 globally as shown in the pie chart (Figure 1-Figure 15) accompanied with the ranking of 45 countries in the order from highest to lowest by the end of 2020 and the current sitution mid-January 2022.
