# Supplementary material for: Partial-Methylated HeyL Promoter Predicts the Severe Illness in Egyptian COVID-19 Patients
Source: Dis Markers. 2022 May 31;2022:6780710. doi: 10.1155/2022/6780710 (PMC9153385; doi:10.1155/2022/6780710)
Supplement: Supplementary Materials — Supplementary 1: Powerpoint showed the presentation of the analysis of data published by the WHO [1] of the prevalence, incidence, and deaths related to the spreading of COVID-19 globally as shown in the pie chart (Figure 1-Figure 15) accompanied with the ranking of 45 countries in the order from highest to lowest by the end of 2020 and the current sitution mid-January 2022. [file 6780710.f1.pdf]

Data analysis of the COVID-19 prevalence, incidence and death in Egypt vs most of countries by the end of first wave (Dec. 2020) to date (mid Jan. 2022)

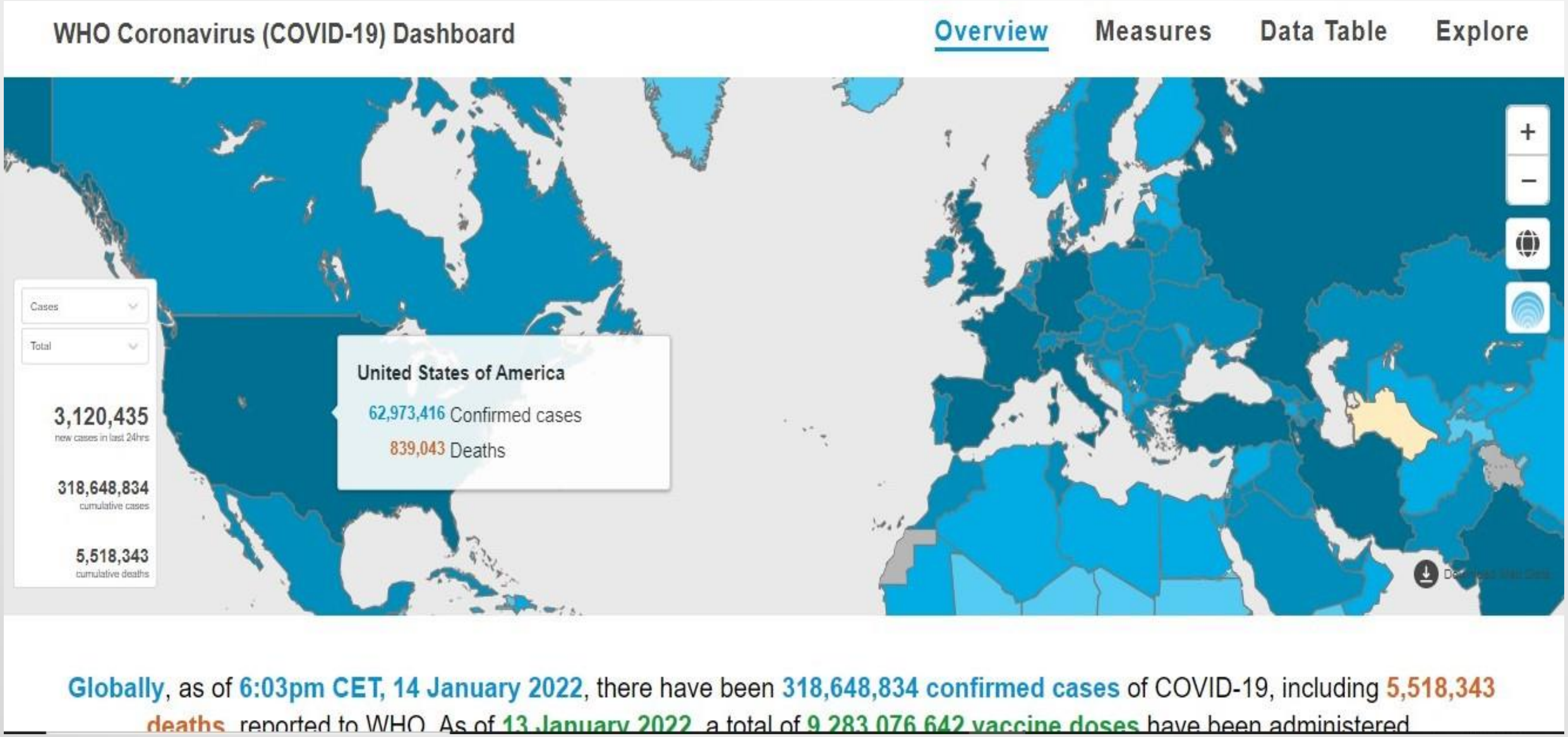

Figure 1: presenting confirmed cumulative cases of COVID-19 and related deaths in USA by mid Jan. 2022

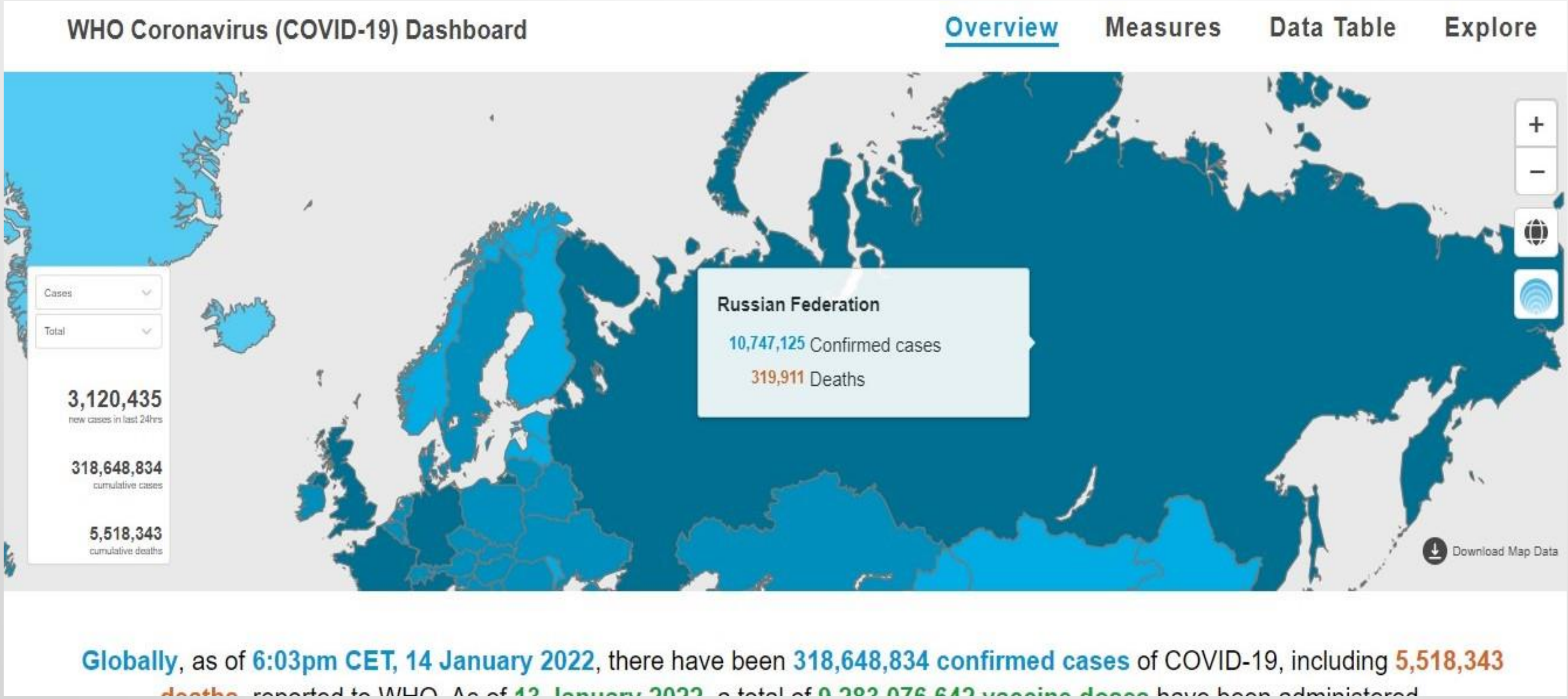

Figure 2: presenting confirmed cumulative cases of COVID-19 and deaths in Russia by mid Jan. 2022

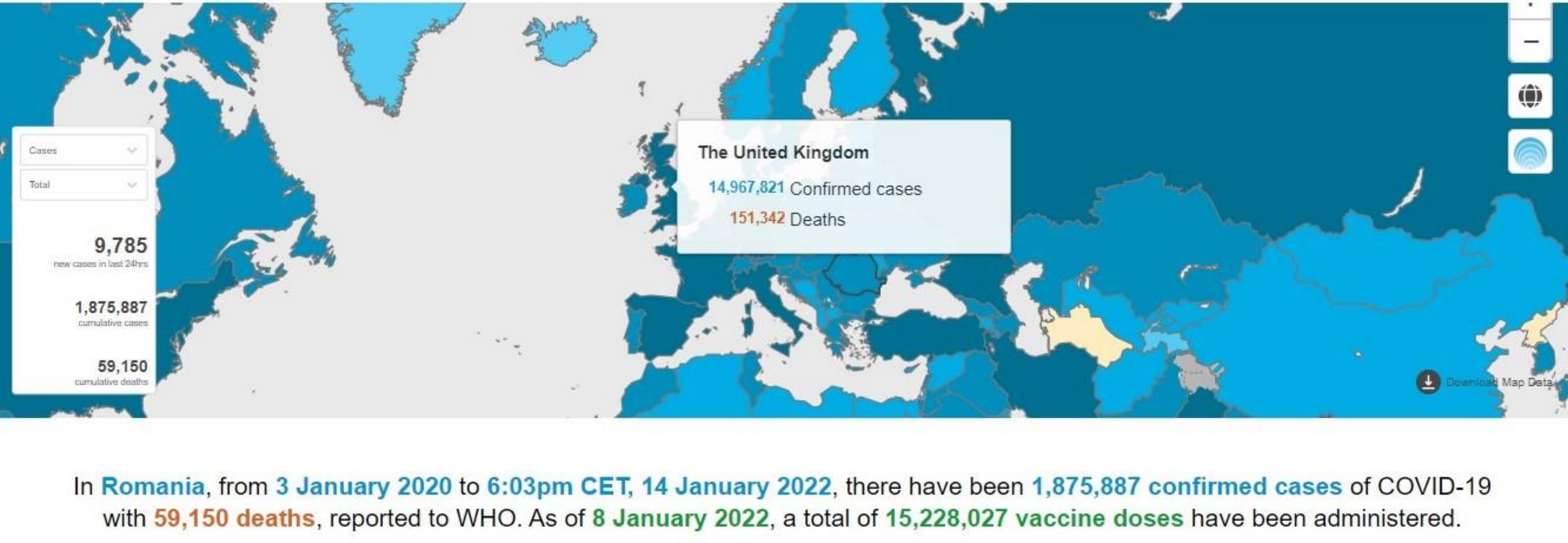

Figure 3: presenting confirmed cumulative cases of COVID-19 and deaths in United Kingdom by mid Jan. 2022

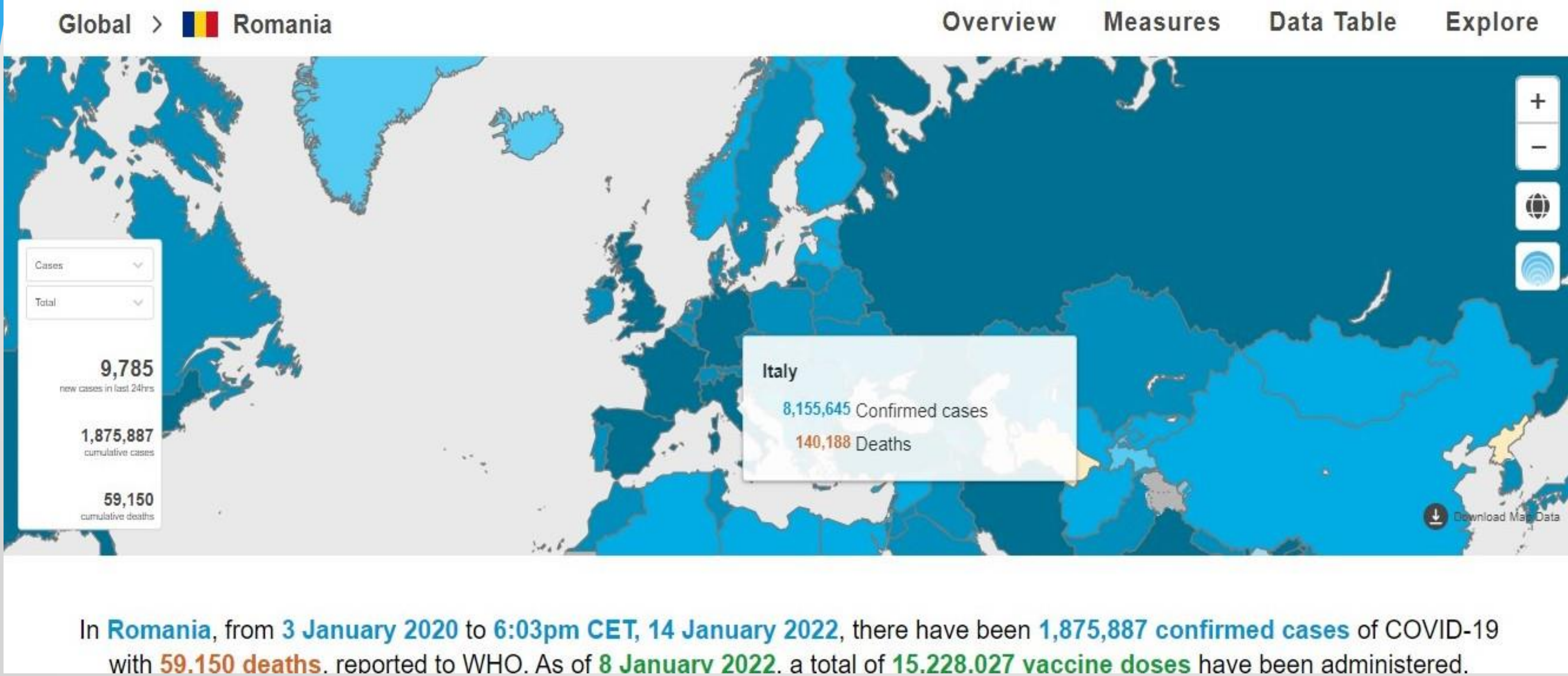

Figure 4: presenting confirmed cumulative cases of COVID-19 and deaths in Italy by mid Jan. 2022.

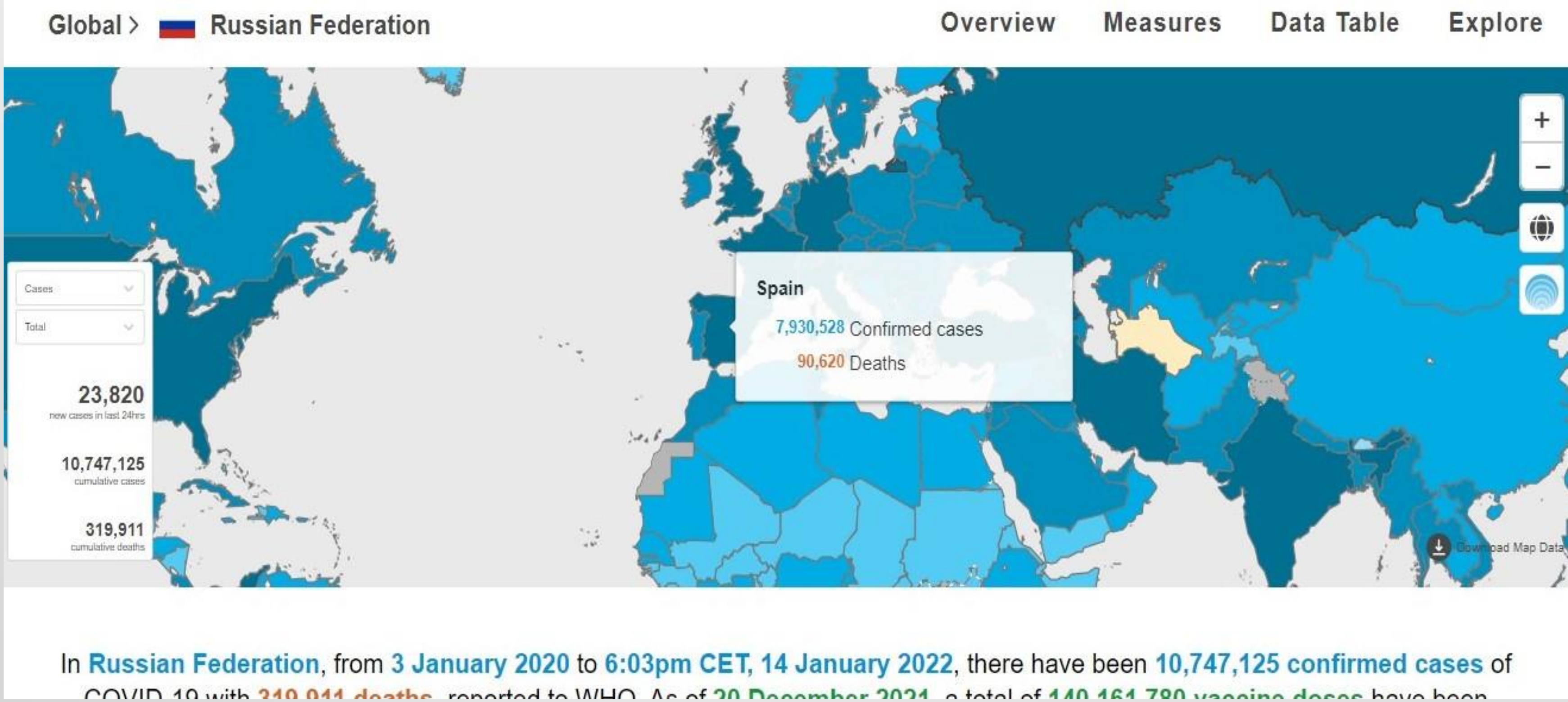

Figure 5: presenting confirmed cumulative cases of COVID-19 and deaths in Spain by mid Jan. 2022.

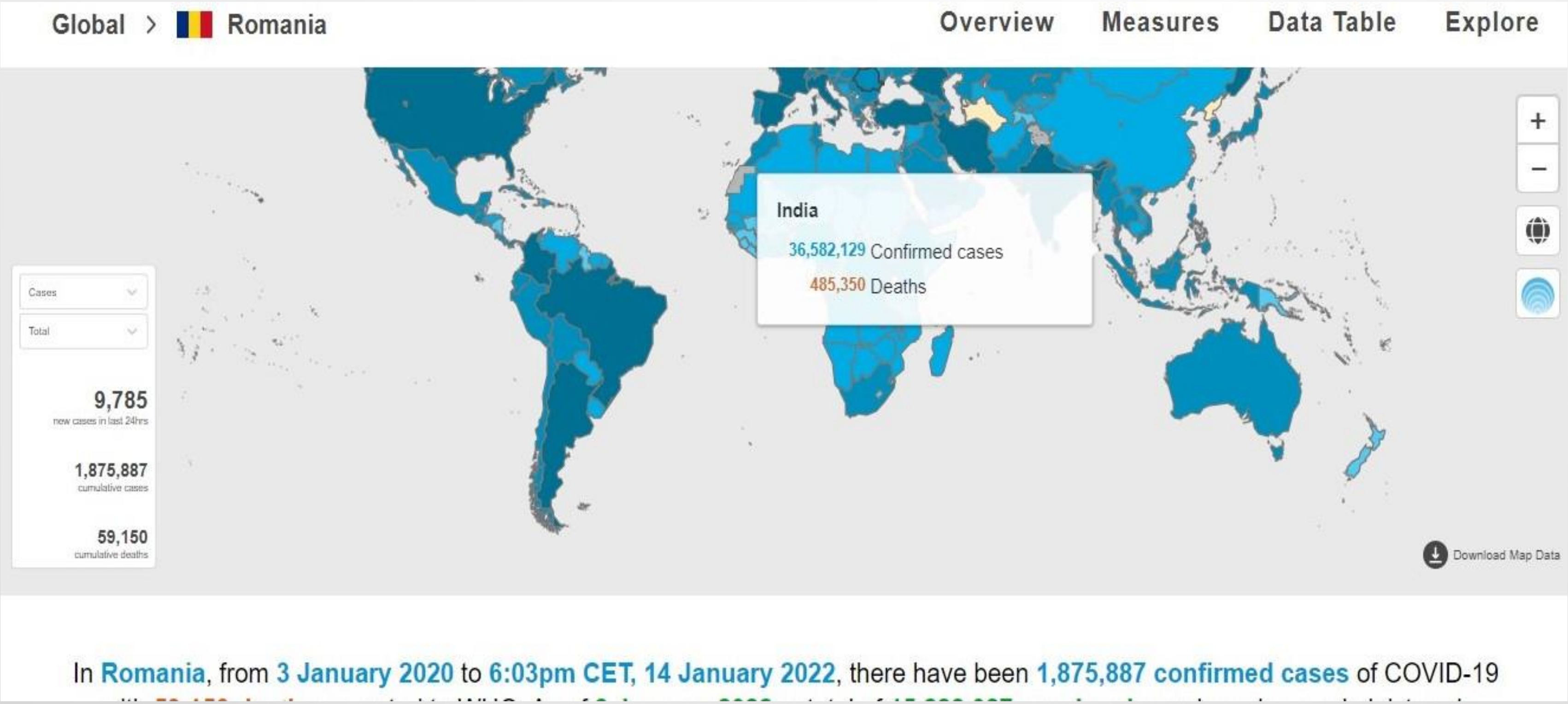

Figure 6: presenting confirmed cumulative cases of COVID-19 and deaths in India by mid Jan. 2022.

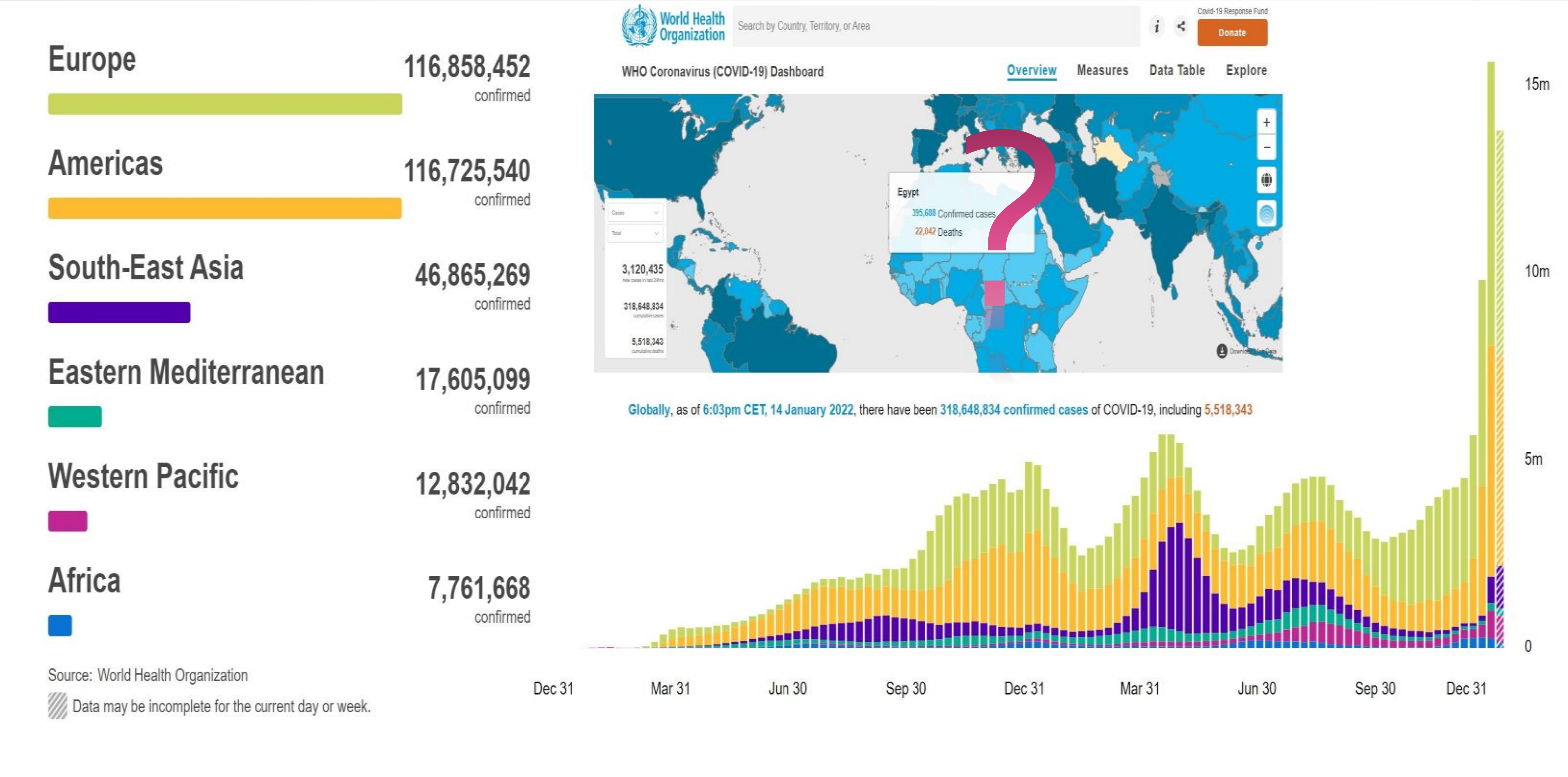

Figure 7: presenting confirmed cumulative cases of COVID-19 and related deaths in Egypt compared to the that worldwide (upper right), graphical presentation of COVID-19 cases among different continents to the end of 2021 (lower right), confirmed cumulative cases of COVID-19 in different continents by January 2022(left side)

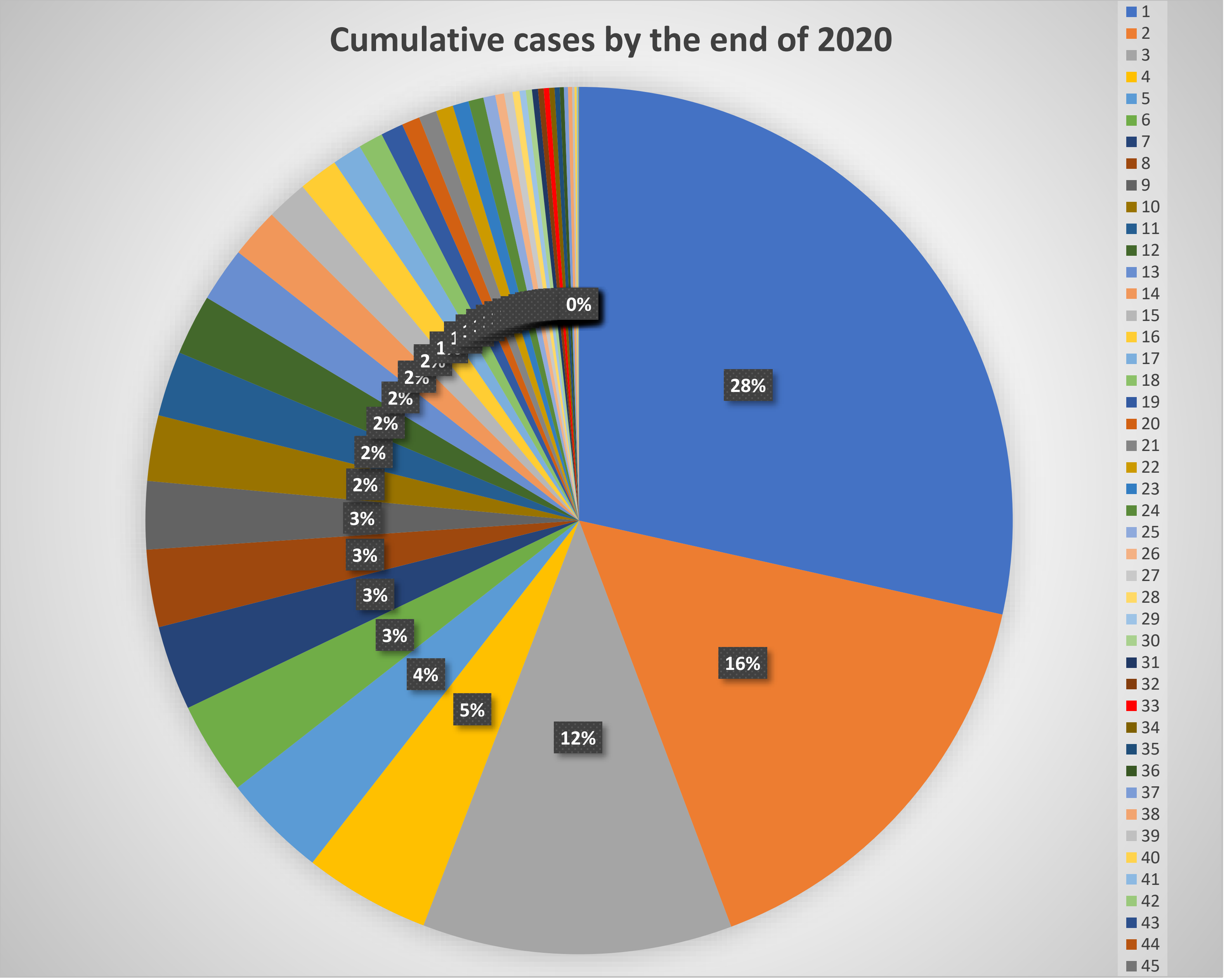

		Cumulative_[illegible]ases
1	USA	18311405
2	India	10169118
3	Brazil	7425593
4	Russia	3021964
5	France	2505074
6	England	2221316
7	Italy	2028354
8	Spain	1854951
9	Germany	1627103
10	Argentine	1571680
11	Colombia	1559766
12	mexico	1450679
13	Turkey	1303917
14	Iran	1189203
15	South Africa	983359
16	poland	942549
17	indonsia	706837
18	Iraq	589943
19	Canada	539298
20	switzerland	433002
21	Morocco	428193
22	sweden	412370
23	Israel	383715
24	Saudi	361903
25	Jordan	285306
26	Japan	213547
27	Emirates	199665
28	Lebanon	168069
29	Kuwait	149277
30	Palestine	147234
31	Qatar	142734
32	Greece	134852
**33**	**Egypt**	**130126**
34	Tunisia	128578
35	Ethiopia	121880
36	Libya	97653
37	Algeria	97441
38	China	96240
39	Singapore	58509
40	Afghanstain	51764
41	Australia	28296
42	Angola	17099
43	Somalia	4690
44	South Sudan	3308
45	Nigeria	2803

Figure 8: pie chart presenting the percentage of cumulative cases of COVID-19 cases among the studied countries by the end of 2020 (left), ranking of cumulative cases showing the order of Egypt that's written in red (right).

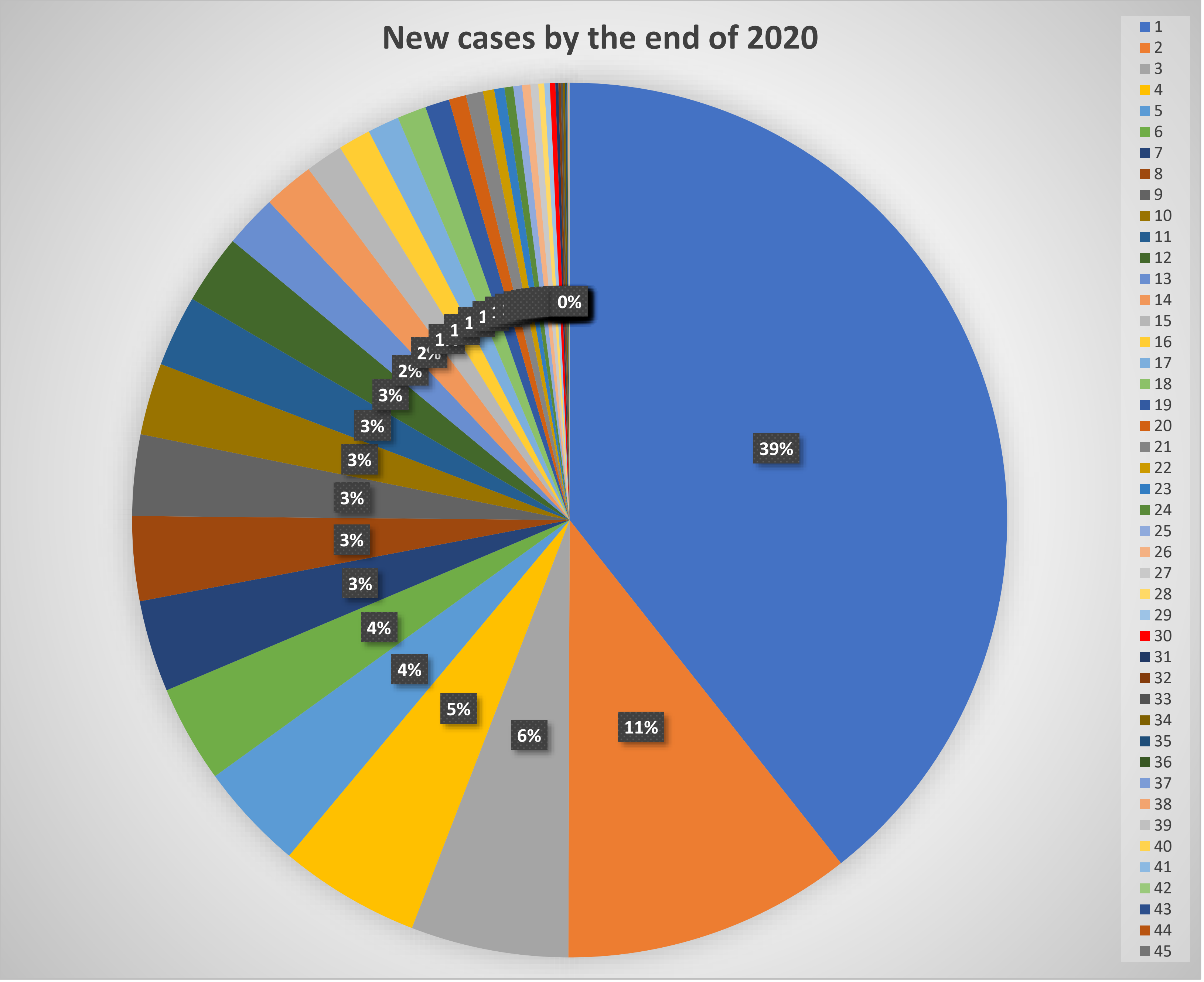

		New_cases
1	USA	221145
2	Brazil	60076
3	England	32725
4	Russia	29258
5	India	22273
6	France	20199
7	Italy	19037
8	Turkey	17543
9	Poland	16846
10	Colombia	14940
11	South Africa	14796
12	Germany	14455
13	Canada	10944
14	Mexico	10480
15	Argentine	7815
16	Indonsia	6740
17	Spain	6581
18	Iran	6021
19	Sweden	5036
20	Japan	3567
21	Israel	3563
22	Morocco	2329
23	Lebanon	2136
24	Tunisia	1826
25	Palestine	1812
26	Switzerland	1721
27	Jordan	1616
28	Emirates	1230
29	Iraq	1140
30	Egypt	1133
31	Greece	617
32	Ethiopia	481
33	Libya	461
34	Algeria	434
35	Kuwait	260
36	Saudi	178
37	Afghanstain	169
38	Qatar	129
39	China	81
40	Angola	70
41	Australia	34
42	Singapore	14
43	Somalia	0
44	South Sudan	0
45	Nigeria	0

Figure 9: pie chart presenting the percentage of new cases of COVID-19 among the studied countries by the end of 2020 (left), ranking of new cases showing the order of Egypt that's written in red (right).

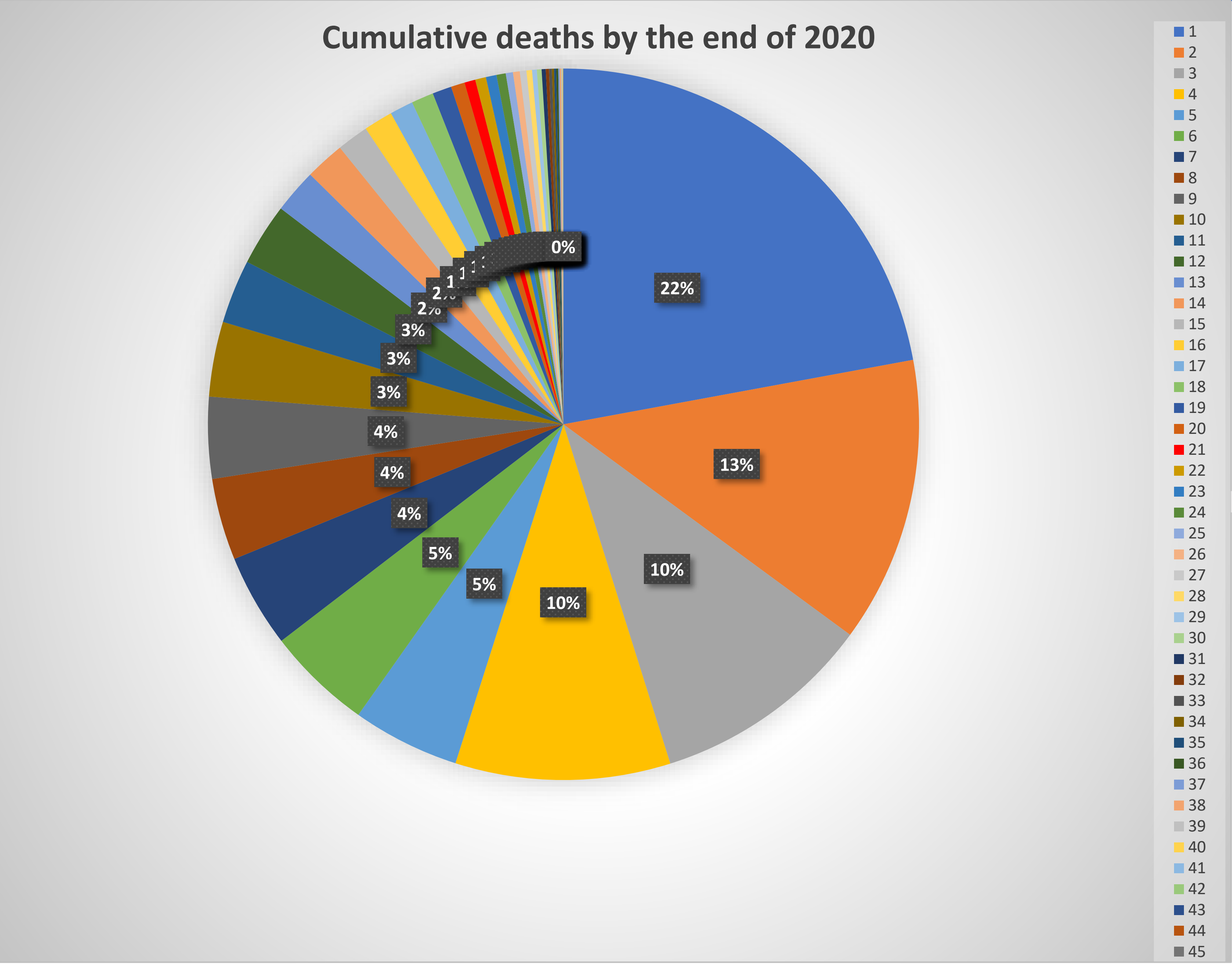

		Cumulative_deaths
1	USA	323527
2	Brazil	190006
3	India	147343
4	Mexico	143054
5	Italy	71359
6	England	70195
7	France	62051
8	Iran	54440
9	Russia	54226
10	Spain	49824
11	Argentine	42392
12	Colombia	41454
13	Germany	29422
14	South Africa	26276
15	Indonsia	20994
16	Turkey	19371
17	Poland	15592
18	Canada	14781
19	Iraq	12755
20	Sweden	9166
**21**	**Egypt**	**7309**
22	Morocco	7170
23	Switzerland	7075
24	Saudi	6168
25	China	4777
26	Greece	4507
27	Tunisia	4385
28	Jordan	3711
29	Japan	3155
30	Israel	3138
31	Algeria	2716
32	Afghanstain	2148
33	Ethiopia	1897
34	Libya	1415
35	Palestine	1385
36	Lebanon	1367
37	Kuwait	926
38	Australia	908
39	Emirates	653
40	Angola	396
41	Qatar	244
42	Somalia	127
43	Nigeria	89
44	South Sudan	62
45	Singapore	29

Figure 10: pie chart presenting the percentage of cumulative deaths related to COVID-19 among the studied ountries by the end of 2020 (left), ranking of cumulative deaths showing the order of Egypt that's written in red

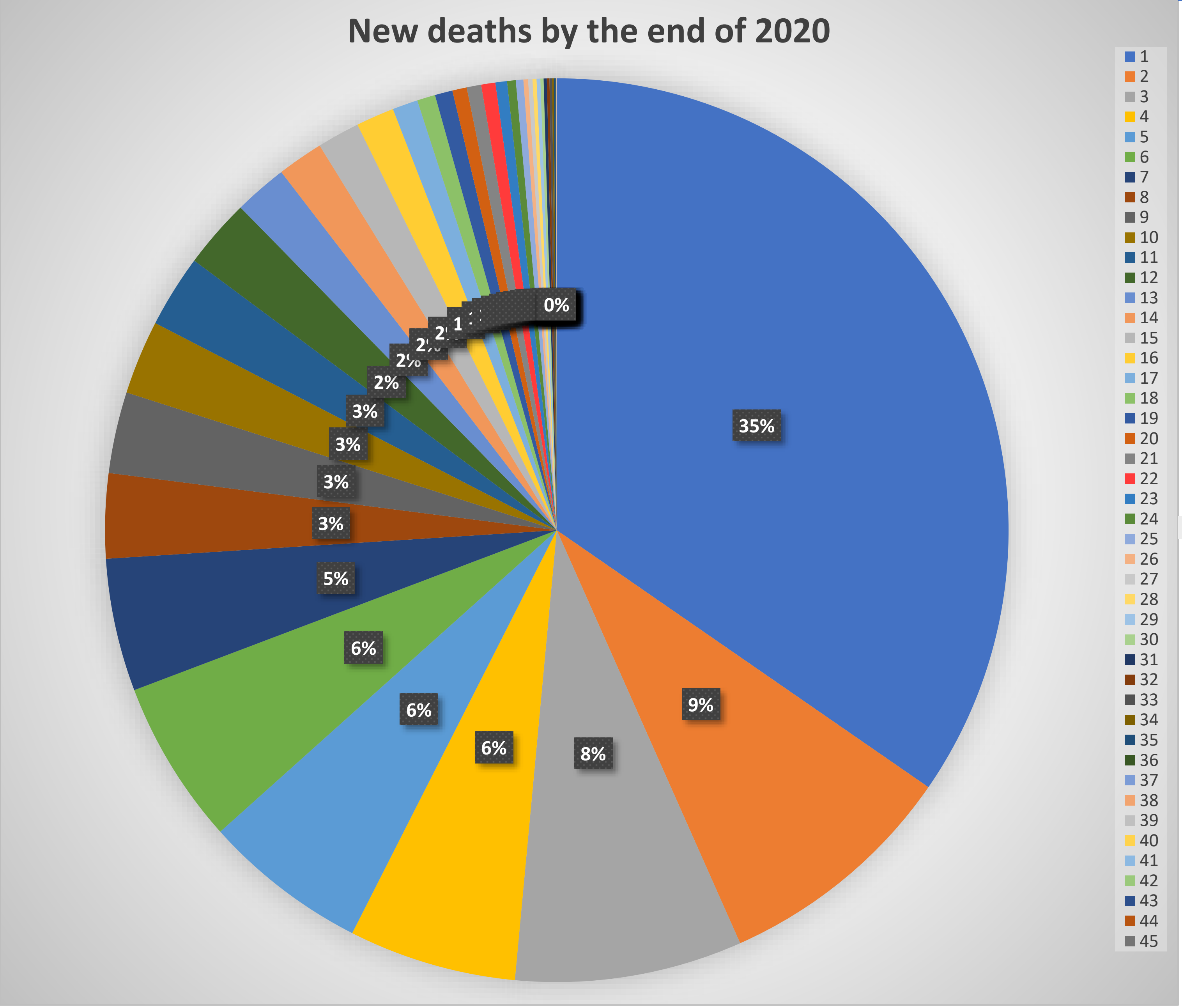

	country	New_deaths
1	USA	3347
2	Mexico	843
3	Brazil	786
4	Poland	580
5	England	570
6	Russia	567
7	Italy	459
8	South Africa	293
9	Colombia	281
10	Turkey	256
11	India	251
12	Germany	240
13	Canada	184
14	France	159
15	Indonsia	147
16	Iran	132
17	Sweden	88
18	Switzerland	63
19	Tunisia	61
20	Greece	50
21	Japan	50
**22**	**Egypt**	**49**
23	Morocco	40
24	Jordan	30
25	Spain	26
26	Libya	16
27	Ethiopia	15
28	Lebanon	14
29	Afghanstain	13
30	Iraq	11
31	Algeria	11
32	Saudi	9
33	Albania	9
34	Emirates	6
35	Israel	6
36	China	1
37	Qatar	1
38	Angola	1
39	Qatar	1
40	Somalia	0
41	South Sudan	0
42	Kuwait	0
43	Singapore	0
44	Nigeria	0
45	Australia	0

Figure 11: pie chart presenting the percentage of new deaths related to COVID-19 among the studied countries by the end of 2020 (left), ranking of new deaths showing the order of Egypt that's written in red (right).

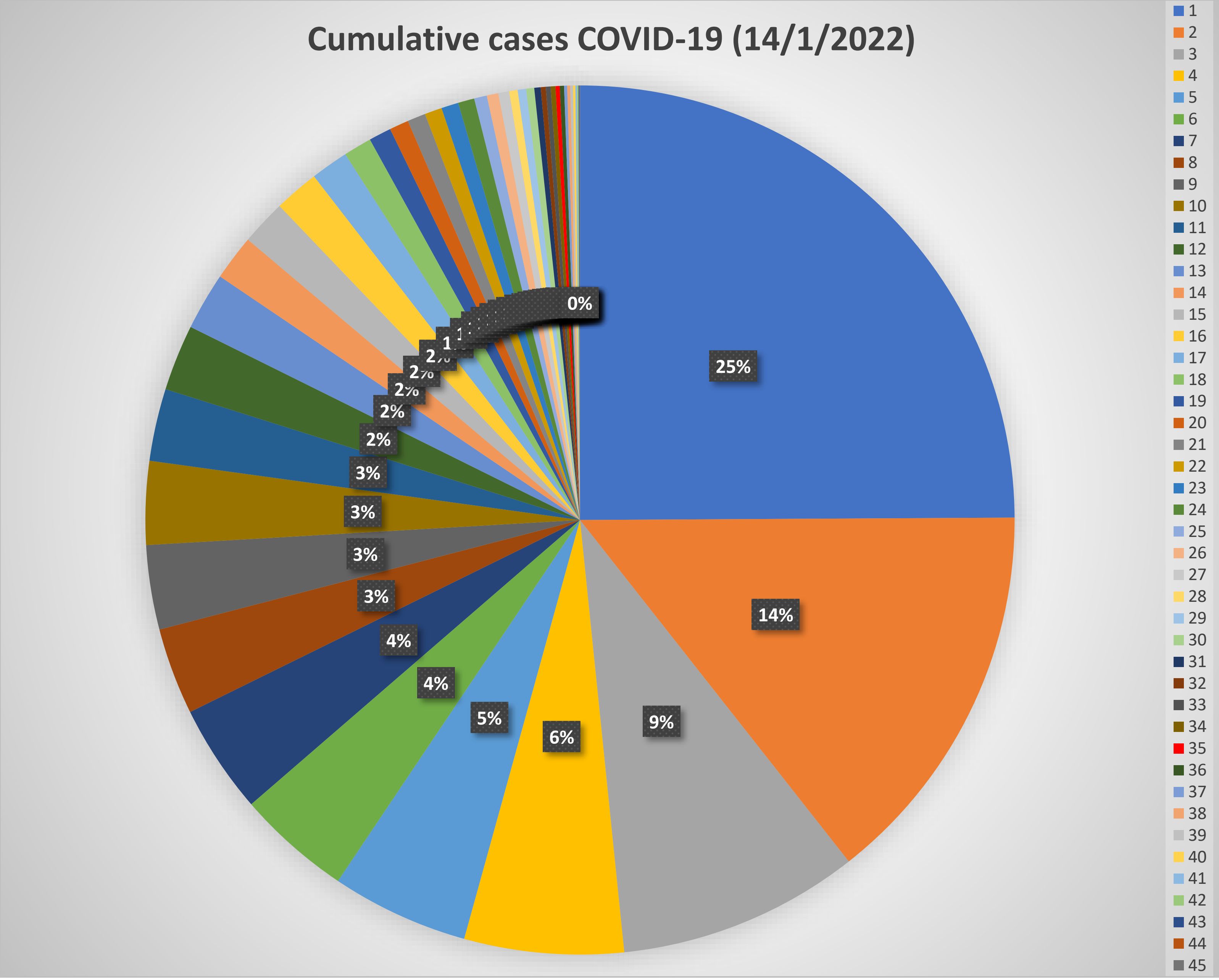

	Country	14/1/2021 Cumulative _cases COVID-19
1	USA	62973416
2	India	36582129
3	Brazil	22716091
4	England	14967821
5	France	12903125
6	Russia	10747125
7	Turkey	10270349
8	Italy	8155645
9	Spain	7930528
10	Germany	7835451
11	Argentine	6793119
12	Iran	6214781
13	Colombia	5410698
14	Poland	4281482
15	Indonsia	4269740
16	Mexico	4214253
17	South Africa	3546808
18	Canada	2657384
19	Iraq	2104940
20	Japan	1809010
21	Israel	1708920
22	Switzerland	1627238
23	Greece	1612869
24	Sweden	1534797
25	Australia	1195158
26	Jordan	1088329
27	Morcco	1017560
28	Emirates	795997
29	Lebanon	794744
30	Tunsia	756155
31	Saudi	599044
32	Palestine	474234
33	Ethiopia	453128
34	Kuawit	451430
35	Egypt	395688
36	Libya	395687
37	Singopore	289085
38	Qatar	287091
39	Nigeria	250009
40	Algeria	224383
41	Afghanistan	158602
42	China	135299
43	Angola	92581
44	Somalia	24261
45	south sudan	16454

Figure 12: pie chart presenting the percentage of cumulative cases of COVID-19 among the studied countries by mid of Jan. 2022 (left), ranking of cumulative cases showing the order of Egypt that's written in red (right).

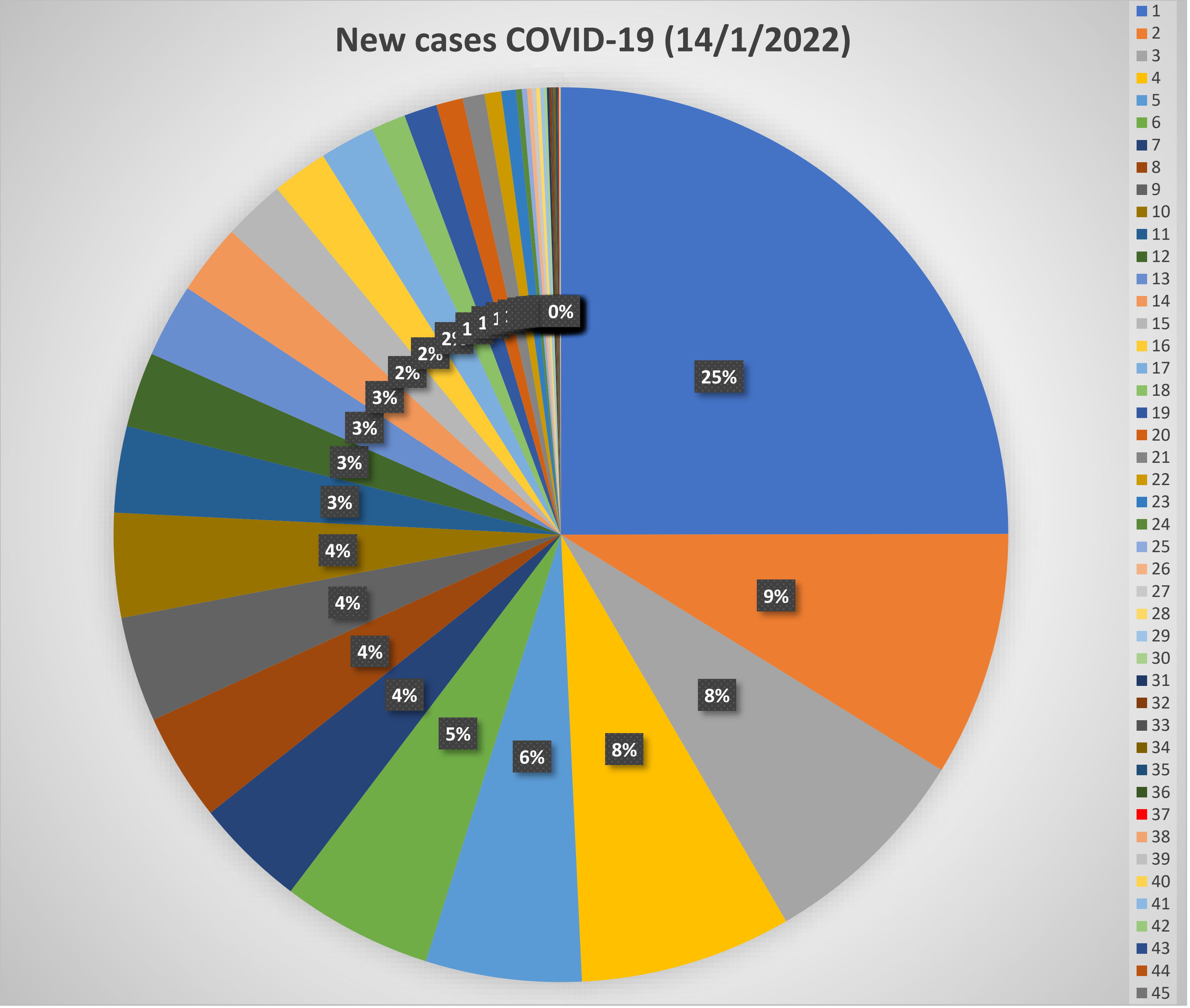

	Country	New_cases COVID-19
1	USA	847054
2	France	299803
3	India	264202
4	Argentine	259484
5	Colombia	191065
6	Italy	184577
7	Sweden	135545
8	Switzerland	132024
9	Russia	129090
10	Mexico	127803
11	England	105679
12	Germany	92223
13	Poland	90289
14	Brazil	86631
15	Turkey	75564
16	Australia	68250
17	Spain	67610
18	South Africa	42254
19	Israel	40430
20	Canada	32597
21	Qatar	26808
22	Greece	20409
23	Japan	17940
24	Lebanon	7246
25	Tunsia	6323
26	Morcco	5518
27	Saudi	5499
28	Kuawit	4883
29	Indonsia	4553
30	Algeria	3585
31	Jordan	2829
32	Emirates	2683
33	Iran	2394
34	Iraq	2385
35	Ethiopia	2131
36	Singopore	960
**37**	**Egypt**	**948**
38	Angola	674
39	Libya	618
40	Palestine	501
41	Nigeria	423
42	China	280
43	Afghanistan	91
44	south sudan	47
45	Somalia	0

Figure 13: pie chart presenting the percentage of new cases of COVID-19 among the studied countries by mid of Jan. 2022 (left), ranking of new cases showing the order of Egypt that's written in red (right).

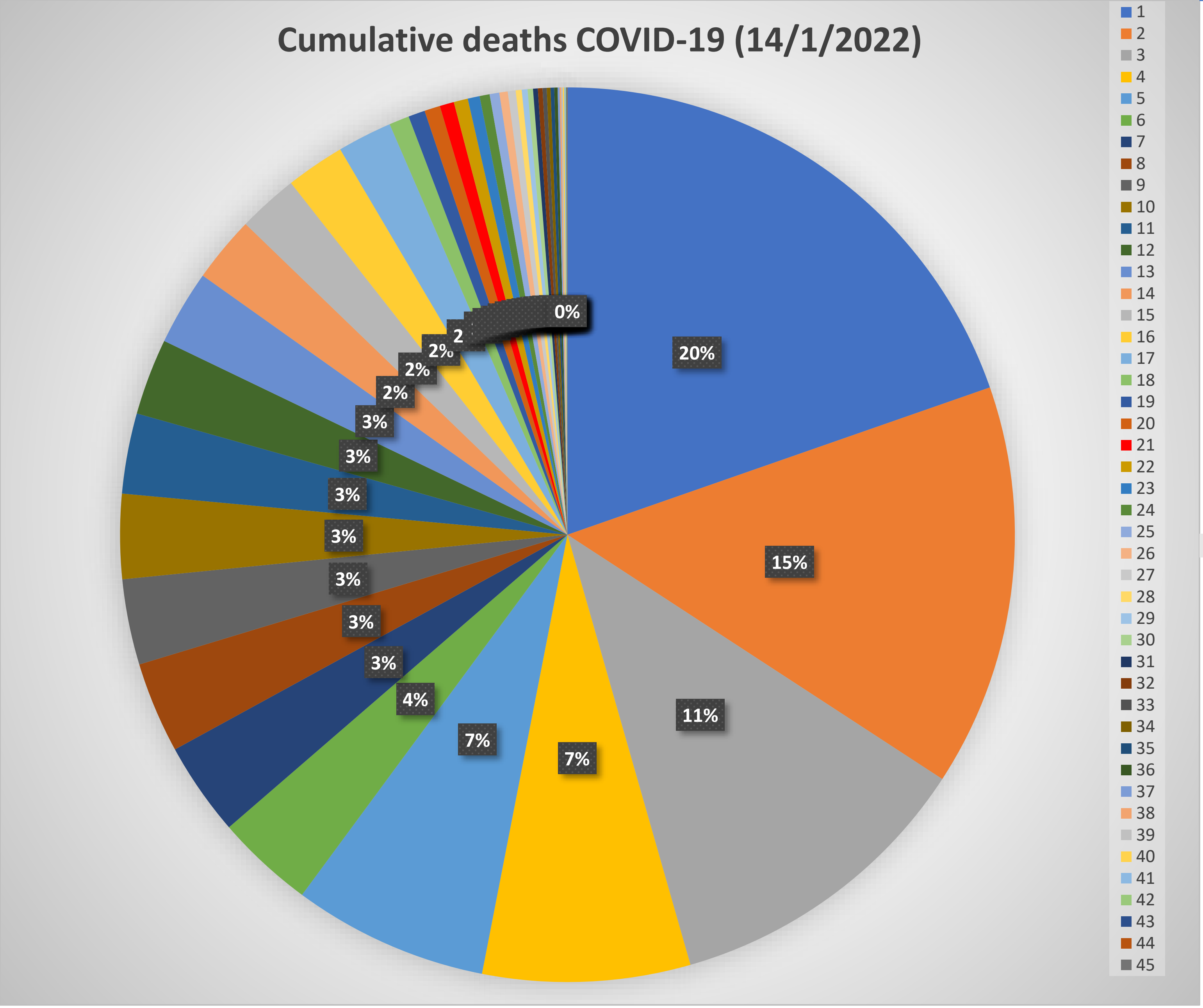

	Country	Cumulative_ deaths COVID-19
1	USA	839043
2	Brazil	620371
3	India	485350
4	Russia	319911
5	Mexico	300764
6	England	151342
7	Indonsia	144163
8	Italy	140188
9	Iran	132002
10	Colombia	130529
11	France	123893
12	Argentine	117808
13	Germany	115337
14	Poland	101841
15	South Africa	92989
16	Spain	90620
17	Turkey	84278
18	Canada	31082
19	Tunsia	25731
20	Iraq	24229
**21**	**Egypt**	**22042**
22	Greece	21732
23	JAPAN	18414
24	Sweden	15482
25	Morcco	14935
26	Jordan	12932
27	Switzerland	12037
28	Lebanon	9325
29	Saudi	8901
30	Israel	8293
31	Afghanistan	7376
32	Ethiopia	7109
33	Algeria	6383
34	Libya	5822
35	China	5700
36	Palestine	5005
37	Nigeria	3092
38	Australia	2522
39	Kuawit	2474
40	Emirates	2182
41	Angola	1847
42	Somalia	1335
43	Singopore	839
44	Qatar	623
45	south sudan	136

Figure 14: pie chart presenting the percentage of cumulative deaths related to COVID-19 among the studied countries by mid of Jan. 2022 (left), ranking of cumulative deaths showing the order of Egypt that's written in red (right).

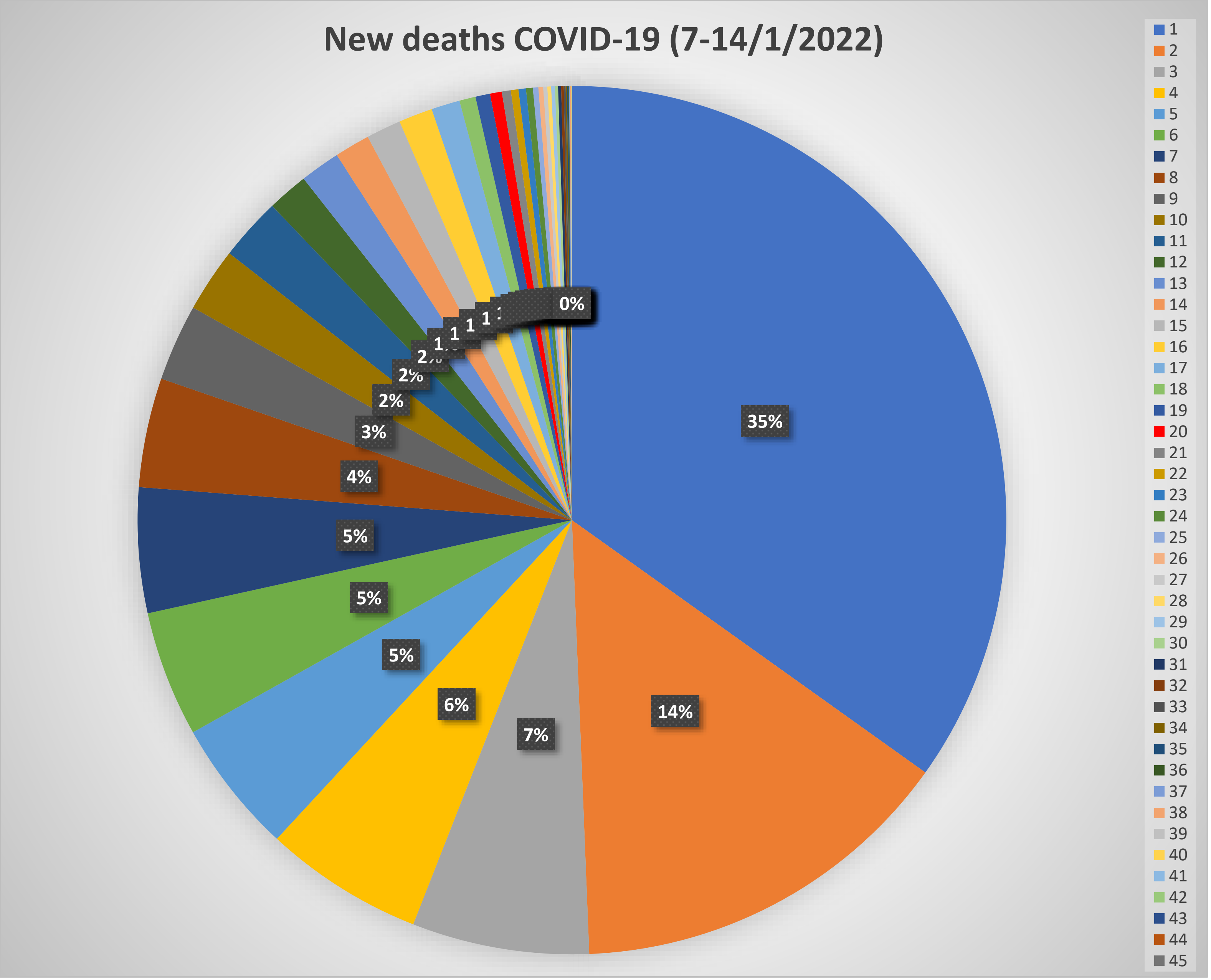

	Country	New_deaths COVID-19
1	USA	12769
2	Russia	5307
3	Poland	2413
4	India	2172
5	England	1827
6	Italy	1714
7	Germany	1705
8	France	1490
9	Turkey	1047
10	south Africa	877
11	Brazil	858
12	Canada	558
13	Greece	547
14	Mexico	482
15	Spain	474
16	Argentina	462
17	Colombia	389
18	Australia	221
19	Iran	200
20	Egypt	160
21	Jordan	122
22	Ethiopia	104
23	Tunisia	101
24	Lebanon	94
25	Switzerland	74
26	Algeria	65
27	Libya	55
28	Morcco	52
29	Angola	51
30	Indonsia	42
31	Sweden	41
32	Iraq	35
33	Israel	26
34	south sudan	18
35	Japan	16
36	Saudi	13
37	Emirates	12
38	Afghanistan	10
39	Kuawit	5
40	Qatar	5
41	Singopore	4
42	China	1
43	Palestine	0
44	Nigeria	0
45	Somalia	0

Figure 15: pie chart presenting the percentage of new deaths related to COVID-19 among the studied countries by mid of Jan. 2022 (left), ranking of new deaths showing the order of Egypt that's written in red (right).
